# Large expansion of oil industry in the Ecuadorian Amazon: biodiversity vulnerability and conservation alternatives

**DOI:** 10.1002/ece3.2099

**Published:** 2016-06-24

**Authors:** Janeth Lessmann, Javier Fajardo, Jesús Muñoz, Elisa Bonaccorso

**Affiliations:** ^1^ Departamento de Ecología Facultad de Ciencias Biológicas Pontificia Universidad Católica de Chile Alameda 340 8331150 Santiago Chile; ^2^ Instituto de Ecología y Biodiversidad (IEB) Casilla 653 Santiago Chile; ^3^ Centro de Investigación de la Biodiversidad y Cambio Climático Universidad Tecnológica Indoamérica Machala y Sabanilla Cotocollao EC170103 Quito Ecuador; ^4^ Centro Universitario de Mérida Universidad de Extremadura C/Santa Teresa de Jornet 38 06800 Mérida Spain; ^5^ Real Jardín Botánico (RJB‐CSIC) Plaza de Murillo 2 28014 Madrid Spain; ^6^ Biodiversity Institute University of Kansas Lawrence Kansas

**Keywords:** Ecosystem diversity, environmental policy, priority conservation areas, species distribution model, species diversity, systematic conservation planning

## Abstract

Ecuador will experience a significant expansion of the oil industry in its Amazonian region, one of the most biodiverse areas of the world. In view of the changes that are about to come, we explore the conflicts between oil extraction interests and biodiversity protection and apply systematic conservation planning to identify priority areas that should be protected in different oil exploitation scenarios. First, we quantified the current extent of oil blocks and protected zones and their overlap with two biodiversity indicators: 25 ecosystems and 745 species (whose distributions were estimated via species distribution models). With the new scheme of oil exploitation, oil blocks cover 68% (68,196 km^2^) of the Ecuadorian Amazon; half of it occupied by new blocks open for bids in the southern Amazon. This region is especially vulnerable to biodiversity losses, because peaks of species diversity, 19 ecosystems, and a third of its protected zones coincide spatially with oil blocks. Under these circumstances, we used Marxan software to identify priority areas for conservation outside oil blocks, but their coverage was insufficient to completely represent biodiversity. Instead, priority areas that include southern oil blocks provide a higher representation of biodiversity indicators. Therefore, preserving the southern Amazon becomes essential to improve the protection of Amazonian biodiversity in Ecuador, and avoiding oil exploitation in these areas (33% of the extent of southern oil blocks) should be considered a conservation alternative. Also, it is highly recommended to improve current oil exploitation technology to reduce environmental impacts in the region, especially within five oil blocks that we identified as most valuable for the conservation of biodiversity. The application of these and other recommendations depends heavily on the Ecuadorian government, which needs to find a better balance between the use of the Amazon resources and biodiversity conservation.

## Introduction

The Ecuadorian Amazon is one of the most biodiverse areas in the world, with outstanding richness of amphibians, birds, fishes, reptiles, bats, and trees (Myers et al. 2000; Bass et al. 2010; Jenkins et al. 2013). This region is also home to at least nine indigenous nationalities, including two voluntarily isolated groups, the Tagaeri and the Taromenane (Brackelaire 2006; CONAIE, 2013). However, despite its high biological and cultural diversity, the region is exposed to numerous social and environmental impacts, mainly caused by copper and gold mining, logging, extensive agriculture, cattle ranching, and, especially, oil extraction (Potes 2010; López et al. 2013).

In Ecuador, oil extraction began in the early 1920s, with a significant increase in production since the 1970s, after the discovery of a rich oil field beneath the Amazon rainforest (Center for Economic and Social Rights, 1994). At present, Ecuador produces ~500,000 barrels of oil per day, the vast majority coming from the northern Amazon provinces of Napo, Sucumbíos, and Orellana (Banco Central del Ecuador, 2014). By 2011, oil production was the main income source of Ecuador, representing 38.7% of government revenues, 58% of exports, and 11.3% of the Gross Domestic Product (Secretaría de Hidrocarburos del Ecuador, 2013).

Contracts for the exploitation of oil fields in Ecuador involve the concession of delimited geographic areas called “blocks,” which have a maximum surface of 200,000 ha (Secretaría de Hidrocarburos del Ecuador, 2011). By 2008, Ecuador had three oil blocks in the Pacific Coast, and about 35 oil blocks in the Amazon, some of them operative and others subject to be leased (Finer et al. 2008). These 35 blocks occupied 52,300 km^2^ of the Ecuadorian Amazon by that year, overlapping with protected areas (PA) and ancestral or titled lands of indigenous groups (Finer et al. 2008). In addition, the most species‐rich area for amphibians, birds, mammals, and plants in the western Amazon had only 14% of its surface protected (by Yasuní National Park), whereas 79% was compromised by active or proposed oil concessions (Bass et al. 2010).

There is extensive documentation of direct and indirect environmental impacts caused by oil exploitation in the Ecuadorian Amazon (Kimerling 1991; Rosenfeld et al. 1997; Fontaine 2003; San Sebastian and Hurtig 2004; Bravo 2007; Finer et al. 2008; De la Bastida 2009; Larrea et al. 2010). In Ecuador, this activity has been characterized by the use of obsolete technology and the application of poor environmental controls (Kimerling 1991; Domínguez 2010). For example, only between 1994 and 2001, 29,000 crude oil barrels were spilled across the Ecuadorian Amazon, of which ~7000 were never recovered from the environment (Fontaine 2003). Wastes of diverse composition, including formation water and drilling muds, have been frequently thrown into open ponds, from which they directly discharge into the environment (Kimerling 1991). As a result of the release of billions of gallons of untreated toxic wastes, health problems in local populations (Center for Economic and Social Rights 1994) and degradation of species habitats (Caitlin 2014; Arellano et al. 2015) have been reported.

Moreover, historical evidence indicates that by the 1990s oil development had already played a major role in transforming the Napo region (northern Ecuadorian Amazon) into one of the largest deforestation frontiers of the Amazon region (Myers 1993; Sierra 2000). In fact, a strong positive correlation between oil drilling and deforestation rate in the region has been found (Fontaine 2003). This relationship is explained by a sequence of events: the opening of roads in the rainforest (necessary for establishing and maintaining oil operations) drives widespread colonization, which then drives deforestation and agriculture expansion (Sierra 2000; Bilsborrow et al. 2004).

Impacts of the oil industry have also been documented inside the PA of the Ecuadorian Amazon, with numerous reports of oil spills inside Yasuní National Park and Cuyabeno Wildlife Reserve (Oilwatch & World Rainforest Movement, 2004; El Comercio, 2006a,b; El Universo, 2007; HOY, 2007). In addition, the northwest portion of Yasuní National Park is accessible through the 150‐km Maxus road created to access drilling platforms within the reserve. This road has dramatically increased the encroachment of the area by Kichwa and Huaorani people, hastening the deforestation rate along the road to ~0.11% per year (Fontaine 2003; Greenberg et al. 2005). Also, contact with intercultural settlers has changed indigenous culture patterns, increasing commercial hunting of wildlife to levels that could compromise ecosystem functioning (Suárez et al. 2009; Ponce‐Reyes et al. 2013; Espinosa et al. 2014). Beyond doubt, these and other environmental impacts caused by the oil industry are reducing biodiversity and threatening wildlife (Canady and Rivadeneyra 2001; Fiori and Zalba 2003; Tellkamp et al. 2004; Wildlife Conservation Society, 2006; Suárez et al. 2009; Bass et al. 2010; Espinosa et al. 2014; McCracken and Forstner 2014), which results in alterations to ecosystem functioning (Anderson et al. 2011; Isbell et al. 2011).

It is often argued that oil extraction only affects the specific points destined to operations, such as oil wells and local camps. However, in practice, impacts generated by the oil industry, such as exploration activities, road openings, noise from platforms, and spills of oil and toxic waste into freshwater systems, may affect large areas beyond wells and camps (O'Rourke and Connolly 2003; Colectivo de Geografía Crítica de Ecuador, 2014). Consequently, oil blocks are considered as spatial units where the environment, the biodiversity, and the health of local populations are potentially vulnerable.

Despite the high proportion of the Ecuadorian Amazon that is already concessioned to the oil industry, the Government of Ecuador is planning to intensify oil extraction in the region. In July 2013, Ecuador's president announced the exploitation of the Ishpingo‐Tambococha‐Tiputini (ITT) oil field, which lies beneath an intact, remote section of the Yasuní National Park. From 2007 to 2013, the preservation of this area was an emblematic environmental project of the government: the Yasuní‐ITT Initiative. The project sought to avoid exploiting oil reserves underneath the ITT block, in exchange for international contributions of at least half of the profits that the country would have received in case of exploiting it (Larrea and Warnars 2009). However, when the initiative did not meet the expectations, the program was terminated in favor of the exploitation of the ITT block.

**Figure 1:**
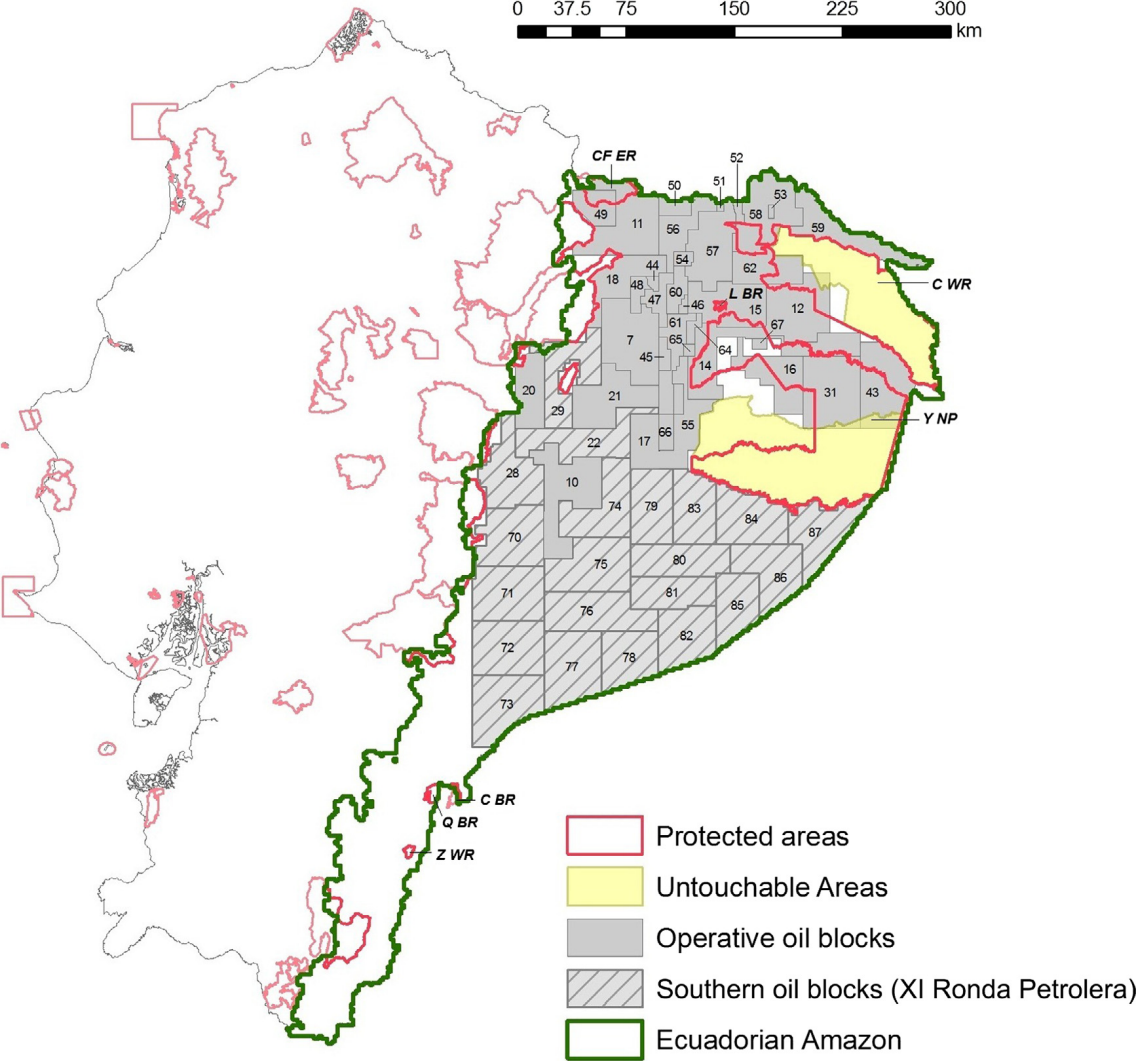
Study area, the Ecuadorian Amazon. Oil blocks (restructured in 2011) and protected zones in the Ecuadorian Amazon. Solid gray indicates blocks already operative. Hashed gray indicates southern oil blocks, which are part of the *XI Ronda Petrolera*. Each oil block shows its identification number, as established by Secretaría de Hidrocarburos del Ecuador (2013). Public protected areas in the Amazon are: Yasuní National Park (Y NP), Cuyabeno Wildlife Reserve (C WR), Limoncocha Biological Reserve (L BR), Cofán Bermejo Ecological Reserve (CB ER), El Quimi Biological Reserve (Q BR), El Cóndor Biological Reserve (C BR), and El Zarza Wildlife Reserve (Z WR).

Besides the extraction of oil within the Yasuní National Park, the Government of Ecuador is promoting the expansion of oil exploitation across the southern Amazon (Fig. 1). In 2011, the Ministry of Hydrocarbons produced the most recent restructuring of the Ecuadorian oil map, resulting in 65 oil blocks, 57 of them in the Amazon (Secretaría de Hidrocarburos del Ecuador, 2013). Three types of changes were incorporated into this new oil map: the split of former larger blocks into smaller blocks; the creation of new blocks on some marginal production fields (<5000 barrels per day) in the north; and the establishment of 21 new blocks in the southern Amazon (Secretaría de Hidrocarburos del Ecuador, 2013). Blocks in the southern Amazon are under a bidding process called *XI Ronda Petrolera*, which began in November 2012. In this bidding process, oil blocks are offered to Petroecuador and Petroamazonas (state‐owned companies), mixed (private–public) companies, and private companies (Secretaría de Hidrocarburos del Ecuador, 2013). To date, offers for only four oil blocks (28, 29, 79, and 83) have been submitted by oil companies, thus a relaunch of the remaining blocks has been discussed (Secretaría de Hidrocarburos del Ecuador, 2013). However, at the end of these negotiations, all blocks not allocated to private or mixed companies will be assigned to Petroamazonas (El Comercio, 2013). Paradoxically, confirmed oil reserves in the southern Amazon are relatively small in comparison to those of other blocks (Secretaría de Hidrocarburos del Ecuador, 2013), whereas the affected area encompasses 30,000 km^2^ of virgin forest (Larrea et al. 2010), holds outstanding biodiversity (Bass et al. 2010; Larrea et al. 2010), and is home to numerous indigenous communities of different nationalities (Larrea et al. 2010; CONFENIAE and CONAIE, 2012).

Addressing the current conflicts between economic interests and land protection in the Ecuadorian Amazon requires objective planning that considers the current deficiencies in biological conservation and its vulnerability, and the importance of using natural resources responsibly. In this context, the field of systematic conservation planning emerges as a proper methodological framework, as it offers structured guides and explicit criteria for reviewing the role of PA in preserving the biodiversity, identifying priority areas to complement the current protection, and devising management policies in different socioeconomic contexts (Margules and Pressey 2000; Sarkar and Illoldi‐Rangel 2010). Systematic conservation planning also provides decision support software to assist the selection of priority areas for conservation that adequately represent the biodiversity with as much economy of resources as possible, being useful for planning in the tropical regions where funds and spaces destined for conservation are often limited (Sarkar and Illoldi‐Rangel 2010). This approach is also convenient for conservation planning in tropical regions, as their complex patterns of species disjunction and co‐occurrence make identification of priority areas for conservation a real challenge (O'Dea et al. 2006).

Given the seemingly irrepressible expansion of the oil industry in the Ecuadorian Amazon, as well as the historical environmental impacts associated to oil extraction in the region, the scientific society must commit to generate and share information that planners may use to discuss the future of this important region. With this goal in mind, this study aims to assess the following: (1) what is the current extent of oil blocks in the Amazonian Ecuador; (2) how do these oil blocks overlap with protected zones and biodiversity; (3) which areas, given their biodiversity value, should be protected from oil activities; and (4) how important is each oil block for biodiversity conservation. Thus, our work seeks to explore potential conflicts between oil extraction and the vulnerability of species and ecosystems, and applies systematic conservation planning to offer informed alternatives that could help finding a better balance between economic growth and the irreplaceable assets that lie over one of the most biodiverse regions of the world.

## Methods

### Study area

For the purposes of this study, we defined the limits of the Ecuadorian Amazon on the basis of the 25 Amazonian ecosystems (Ministerio del Ambiente del Ecuador, 2012), covering 100,234 km^2^. In the Ecuadorian Amazon, there are two main categories of reserves: public PA and untouchable areas (UA) (*Zonas Intangibles*) (Fig. 1). UA were created by presidential decrees to protect biodiversity and cultural values from any extractive and industrial activities (Constitución del Ecuador, 2008). There are two UA in the Ecuadorian Amazon, the Tagaeri‐Taromenane UA, which overlaps partially with Yasuní National Park and with the territories of the two voluntarily isolated indigenous groups (the Tagaeri and the Taromenane), and the Cuyabeno‐Imuya UA, which lies within the Cuyabeno Wildlife Reserve (Brackelaire 2006). Hereby, the set of PA and UA is called “protected zones.” Boundaries of protected and UA are available from Instituto Geográfico Militar (http://www.igm.gob.ec/).

### Biodiversity indicators

We selected terrestrial species and ecosystems as biodiversity indicators and conservation features. The species set was composed of 86 amphibians, 267 birds, 49 heliconiine butterflies, 32 terrestrial mammals of medium and large size, and 311 vascular plants. For describing species distributions within the study area, we used species distribution models (SDMs), which usually provide more realistic outcomes than species geographic ranges (Rondinini et al. 2006; Carvalho et al. 2010), especially at fine geographic scales (Pineda and Lobo 2012). SDMs generalize the empirical relationships between species occurrences and underlying environmental conditions, to predict the probability of species occurrence within a given area (Guisan and Zimmermann 2000). In contrast to point maps and geographic ranges, SDMs improve reliability of species distribution estimates by minimizing both commission (false species presences) and omission errors (false species absences) in the estimated distributions (Bombi et al. 2011). To ensure the use of high quality estimates of species distributions, SDMs were constructed only for species with sufficient occurrence records (≥5) (Hernández et al. 2006), excluding species with high uncertainty and obvious errors in their locality records. All records were obtained from specimen databases of natural history collections (see “Extended Methods” in Appendix S1). SDMs were constructed based on occurrence records and the 19 bioclimatic variables from Worldclim 1.4 (Hijmans et al. 2005). The relationship between occurrence records and bioclimatic variables was analyzed with Maxent 3.3.3e (Phillips et al. 2006) (see “Extended methods” in Appendix S1).

In addition to species distributions, we analyzed the distribution maps of the 25 Amazonian ecosystems (Ministerio del Ambiente del Ecuador, 2012). As such, the analysis included a total of 770 biodiversity indicators: 745 species and 25 ecosystems (Table S2, Appendix S2). Whereas the species data set constitutes a biodiversity surrogate of the entire species richness in the Ecuadorian Amazon – which is not possible to represent completely with currently available data – the ecosystems dataset allows adding a coarse‐level filter that aims to ensure the representation of the habitats of those species not included explicitly in the study (Ardron et al. 2008).

### Current extent of oil blocks and their overlap with protected areas and biodiversity

Digital information about the new configuration of oil blocks was obtained from Secretaría de Hidrocarburos del Ecuador (http://www.hidrocarburos.gob.ec/). This information differentiates “operative oil blocks,” which are in exploitation (extraction or exploration) or leased, from “southern oil blocks,” which represent the expansion of the oil industry in the south of the Ecuadorian Amazon and are offered in concession by the *XI Ronda Petrolera*, or future bidding processes (Fig. 1). In ArcMap 9.3 (ESRI, 2009), we quantified the current extent of oil blocks and protected zones across the Ecuadorian Amazon, and the extent of overlap between both.

Species richness maps for the Ecuadorian Amazon, which show the number of species by ~1 km^2^, were generated for each species group and for all species, by summing all individual SDMs (Guisan and Rahbek 2011). Also, we generated a species richness center map to show the sites with the highest diversity for all species groups (Bass et al. 2010). Using this approach, we selected the top class (upper tertile) of each individual species richness map of the five taxonomic groups (Mateo et al. 2013), and overlaid them spatially. Then, in order to analyze the coincidence of biodiversity and oil blocks, we overlapped the species richness center map and the 25 ecosystems with oil blocks. Furthermore, we analyzed the current protection of ecosystems by overlapping them with the extent of protected zones that do not coincide with oil blocks and deforested areas, according to Socio Bosque (2012).

### Priority areas for conservation in different oil extraction scenarios

Given the historical negative impacts of the oil industry in the environment of the Ecuadorian Amazon (Kimerling 1991; Rosenfeld et al. 1997; Fontaine 2003; San Sebastian and Hurtig 2004; Finer et al. 2008), the expansion of the oil industry could seriously increase the vulnerability of species and ecosystems. According to the principles of the systematic conservation planning (Margules and Pressey 2000), to ensure the persistence of biodiversity in the Ecuadorian Amazon in the long‐term, all the species and ecosystems of this region should have part of their distribution (i.e., be represented) in well preserved areas, protected from the potential impacts of the oil industry. In this context, we identified areas in this region that ensure a representation of all the biodiversity indicators and that should be protected from oil extraction. We call these areas “priority areas for conservation.”

The identification of priority areas for conservation requires the establishment of conservation targets, which indicate the proportion of each species distribution and ecosystem extent that is expected to be accounted for in areas without oil activities and other threats. When these targets are achieved in a conservation area network, the species or ecosystems could be considered non‐vulnerable and well represented. As recommended by Ardron et al. (2008), we performed a sensitivity analysis in which we tested the outcomes of different sets of targets in order to select the one which (1) allows the representation of all biodiversity indicators in conservation areas, (2) favors the increase of protection for the most vulnerable species and ecosystems, and (3) are practically achievable in terms of the extent of the priority areas and resources needed. According to these criteria, species with small distributions (<10,000 km^2^) in the Ecuadorian Amazon were assigned the highest target (i.e., protecting 36% of their distribution), whereas the species with widespread extensions (>75,000 km^2^) were assigned the lowest target (i.e., protecting 4% of their distribution). The same principle was used to estimate conservation targets for ecosystems, using different extension thresholds of <100 km^2^ and >10,000 km^2^, to 36% and 4% targets, respectively. For species and ecosystems with ranges of intermediate size, the target was interpolated linearly between the target extremes. These target extremes were selected through a sensitivity test analysis, in which we first established a target that ranged between 90% and 10% of the species and ecosystem distribution, following Rodrigues et al. (2004). Then, we evaluated the performance of four scenarios that had different targets: 100%, 80%, 60%, and 40% of the initial target. In these scenarios, we searched for priority areas across the entire Ecuadorian Amazon, excluding highly intervened areas and considering the protection of the current reserves. As a result, the 100%, 80%, 60%, and 40% scenarios generated a set of priority areas that, together with protected zones, represent 85%, 70%, 55%, and 38% of the extent of Ecuadorian Amazon, respectively. In addition, the 40% scenario was the only one with a complete achievement of the targets. Therefore, we selected the 40% scenario, which means a 36–4% of the species and ecosystem distributions, because higher targets involve unachievable goals and require unfeasible large conservation areas across the Ecuadorian Amazon.

We used Marxan (Ball et al. 2009) to select priority areas for conservation that ensure both biodiversity representation and protection. Marxan's simulated annealing algorithm selects a set of planning units (PUs) that meets the predefined conservation targets while minimizing the total cost of all PUs (or sites) included in the reserve system. In our study, the Ecuadorian Amazon was divided into square‐shaped PUs of 3.45 km^2^, resulting 29,213 PUs. Also, the cost of each PU was equated to its environmental impact in this region, which allowed us to favor the selection of areas with high ecological integrity, instead of degraded areas. Environmental impact was accounted by developing an Environmental Risk Surface (ERS), which considers clear threats to biodiversity, such as human population density, agriculture and cattle ranching, mining (other than oil extraction), oil wells, dams, roads, and airports (Table S1 in Appendix S1). This process yielded a raster layer where each pixel of ~1 km^2^ had an impact value between 0–100, with 100 being the strongest impact.

The priority areas were identified in three different scenarios. In a first scenario, we searched for priority areas in sites that are not affected by current or future oil extraction and excluded highly impacted areas (affected by agriculture, deforestation, urbanization, etc.). To achieve this target, the search algorithm could not select PUs that fell within oil blocks (operative and southern) and in areas with high environmental impact (>32 environmental impact value from ERS). In a second scenario, we allowed the identification of priority areas coinciding with southern oil blocks. As exploitation of these blocks has not started, the areas they occupy have not experienced significant environmental impacts. Thus, we assumed that it is still possible to redefine the configuration of these oil blocks if priority areas are identified within their current limits. For this scenario, we ran Marxan allowing the inclusion of these blocks but excluding operative blocks and areas with high environmental impact. Finally, in a third scenario we identified priority areas across the entire Ecuadorian Amazon, including all oil blocks. As it is possible that all oil blocks (including the southern blocks) become operative, the aim is to highlight important places for biodiversity representation and the need to implement special conservation actions.

For the three scenarios, we forced PUs within existing protected zones to be included in the solution, considering the proportion of species distributions and ecosystems already protected. We analyzed the Marxan's *summed solution* result, which provides the frequency of selection of each PU in solutions produced across 100 replicates. This procedure indicates how important a planning unit is for creating an efficient reserve system (Ardron et al. 2008). Finally, we considered as priority areas the set of PUs that were selected in 75 or more of the 100 replicates (Game and Grantham 2008).

### Oil block importance for biodiversity conservation

As oil blocks are managed as operative units, to guide future conservation efforts we considered relevant to assess the importance of each oil block in terms of biodiversity conservation. To fulfill this target, we calculated an *oil block importance index* that takes into account the importance of the following aspects within each oil block: (1) species diversity, as a combination of the averages of total species richness and priority species richness (i.e., species endemic to Ecuador and threatened in the global (IUCN, 2014) or national red lists (Valencia et al. 2000; Granizo et al. 2002; Ron et al. 2008; Tirira 2011); (2) ecosystem diversity, as the number of ecosystems; (3) level of land preservation, as the extension of natural vegetation in the block divided by its area, using the deforestation map of Socio Bosque (2012); and (4) presence of important areas for conservation, as the proportion of the block coinciding with priority areas for conservation (obtained from the third Marxan scenario) or with protected zones. Later, for each variable, we arranged oil blocks into four quartiles and we assigned them a score from 1 (bottom quartile) to 4 (top quartile). The importance of each block was obtained by summing the scores of all four variables.

## Results

### What is the current extent of oil blocks, and how much do they overlap with protected areas and biodiversity indicators?

According to the study area defined here, to date, 68,196 km^2^ (~68%) of the Ecuadorian Amazon is covered by oil blocks (Table 1). Specifically, 32% of this region corresponds to 36 operative blocks, and 36% to 21 southern blocks (open for bidding). Protected and UA represent 20% and 12% of the region, respectively, but their extensions overlap significantly, resulting in 22% of Ecuadorian Amazon covered by protected zones. However, more than 30% of the extent of PA and 4% of UA coincide with operative oil blocks (Table 2). Four PA in the Amazon have a high proportion of their extension overlapping with oil blocks. Limoncocha Biological Reserve has a 100% overlap, Cofán Bermejo Ecological Reserve an 84%, Yasuní National Park a 45%, and Cuyabeno Wildlife Reserve a 22%. As a result of these overlaps, only 16% of the Ecuadorian Amazon is actually covered by protected zones free of oil blocks.

**Table 1:** Extent of protected areas (PA), untouchable areas (UA), and operative and southern oil blocks in the Ecuadorian Amazon

Category	Extent (km^2^)	Proportion of the Ecuadorian Amazon (%)
PA	19,929	20
UA	11,949	12
Protected zones (PA + UA)	22,172	22
Operative oil blocks	32,570	32
Southern oil blocks	35,626	36
All oil blocks	68,196	68

**Table 2:** Overlaps of oil blocks and protected zones in the Ecuadorian Amazon

Overlap elements	Extent of overlap (km^2^)	Proportion of overlap (%)
Protected areas (PA) in operative oil blocks	6449	32
PA in southern oil blocks	1	0.01
Untouchable areas (UA) in operative oil blocks	498	4
UA in southern oil blocks	0	0
Total protected zones in oil blocks	6473	29
Yasuní National Park in oil blocks	4598	45
Limoncocha Biological Reserve in oil blocks	28	100
Cofán Bermejo Ecological Reserve in oil blocks	462	84
Cuyabeno Wildlife Reserve in oil blocks	1303	22
El Quimi Biological Reserve in oil blocks	0	0
El Cóndor Biological Reserve in oil blocks	0	0
El Zarza Wildlife Reserve in oil blocks	0	0
Ecuadorian Amazon covered by oil blocks	68,196	68
Ecuadorian Amazon covered by protected zones without oil blocks	15,699	16
Ecuadorian Amazon without protected zones and oil blocks	16,339	16

**Figure 2:**
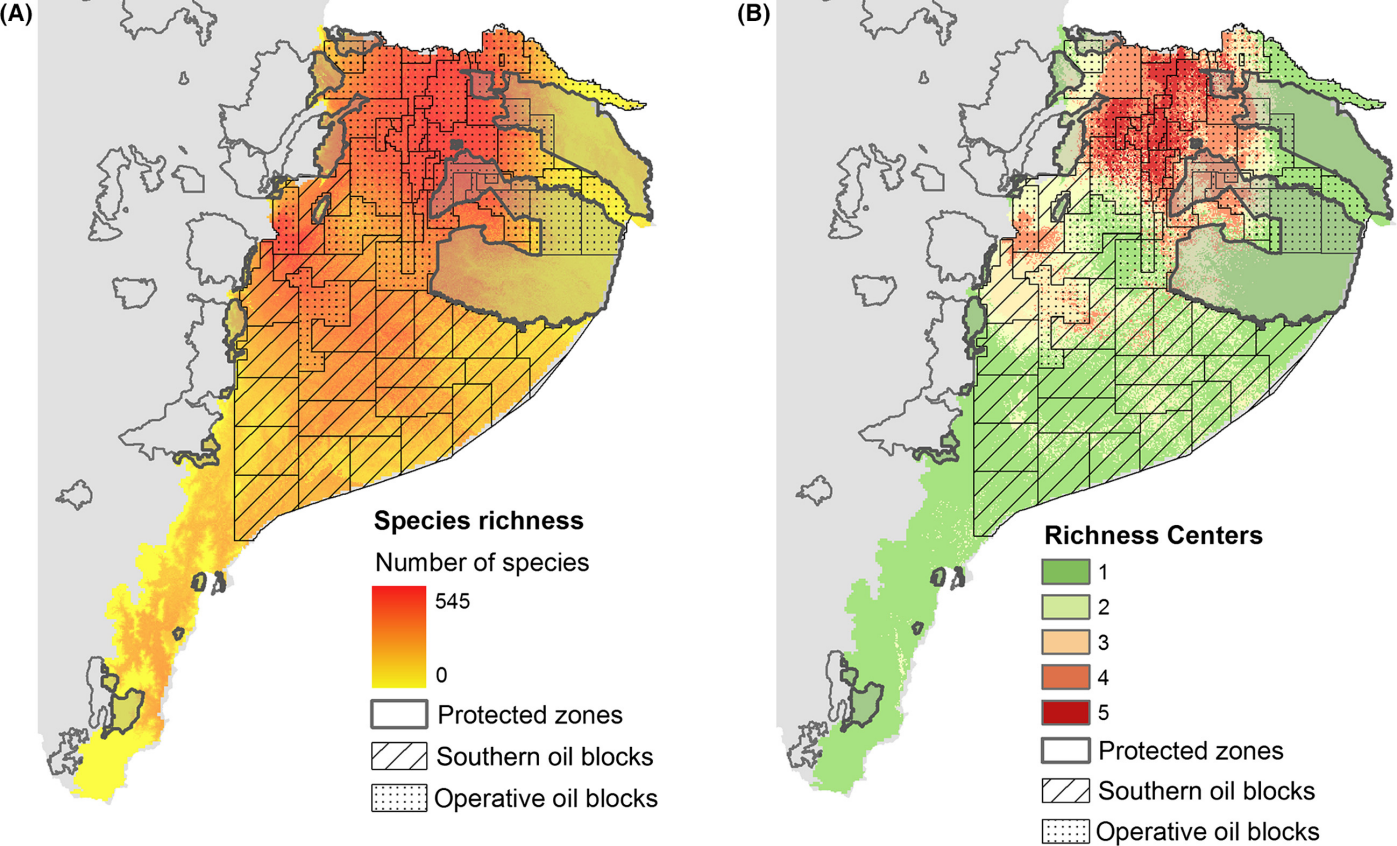
Species richness patterns and richness center of the Ecuadorian Amazon. Species richness map (A) is obtained from the sum of the species distribution models of amphibians, birds, heliconiine butterflies, medium and large terrestrial mammals, and vascular plants. The richness center map of the five key focus groups (B) is observed in the northern Amazon, overlapping with oil blocks.

Regarding biodiversity, the highest species richness areas for amphibians, birds, mammals, heliconiine butterflies, and vascular plants are mainly located in the northern Amazon (Fig. 2A); unfortunately, this is the area where operative oil blocks are concentrated. Moreover, the species richness center of the region, where the highest diversity for all species groups overlap (Fig. 2B), extends 4351 km^2^ and coincides almost completely (99.8%) with oil blocks or areas compromised by deforestation. In addition, the species richness center overlaps with an extremely small percentage of protected zones (0.2%).

Also, we found that 19 of 25 ecosystems in the Ecuadorian Amazon coincide spatially with oil blocks. Of these 19 ecosystems, 12 have more than 50% of their surface covered by oil blocks, and three of them, the “bamboo forest,” the “lowland evergreen forest of the Pastaza,” and the “evergreen forest on sandstone plateau in the Cordillera del Cóndor,” have more than 95% of their surface within oil blocks. Six ecosystems (most of them located in the southern Amazon) are totally outside protected zones; these are “bamboo forest,” “lowland evergreen forest of the Pastaza,” “semideciduous piemontano forest of the South Eastern Andes,” “foothill evergreen forest on limestone outcrops,” “evergreen forest on sandstone plateau in the Cordillera del Cóndor,” and “foothill evergreen forest on sandstone plateaus of the Cordillera del Cóndor‐Kutucú.”

### Which areas should be protected from oil activity given their biodiversity value?

**Figure 3:**
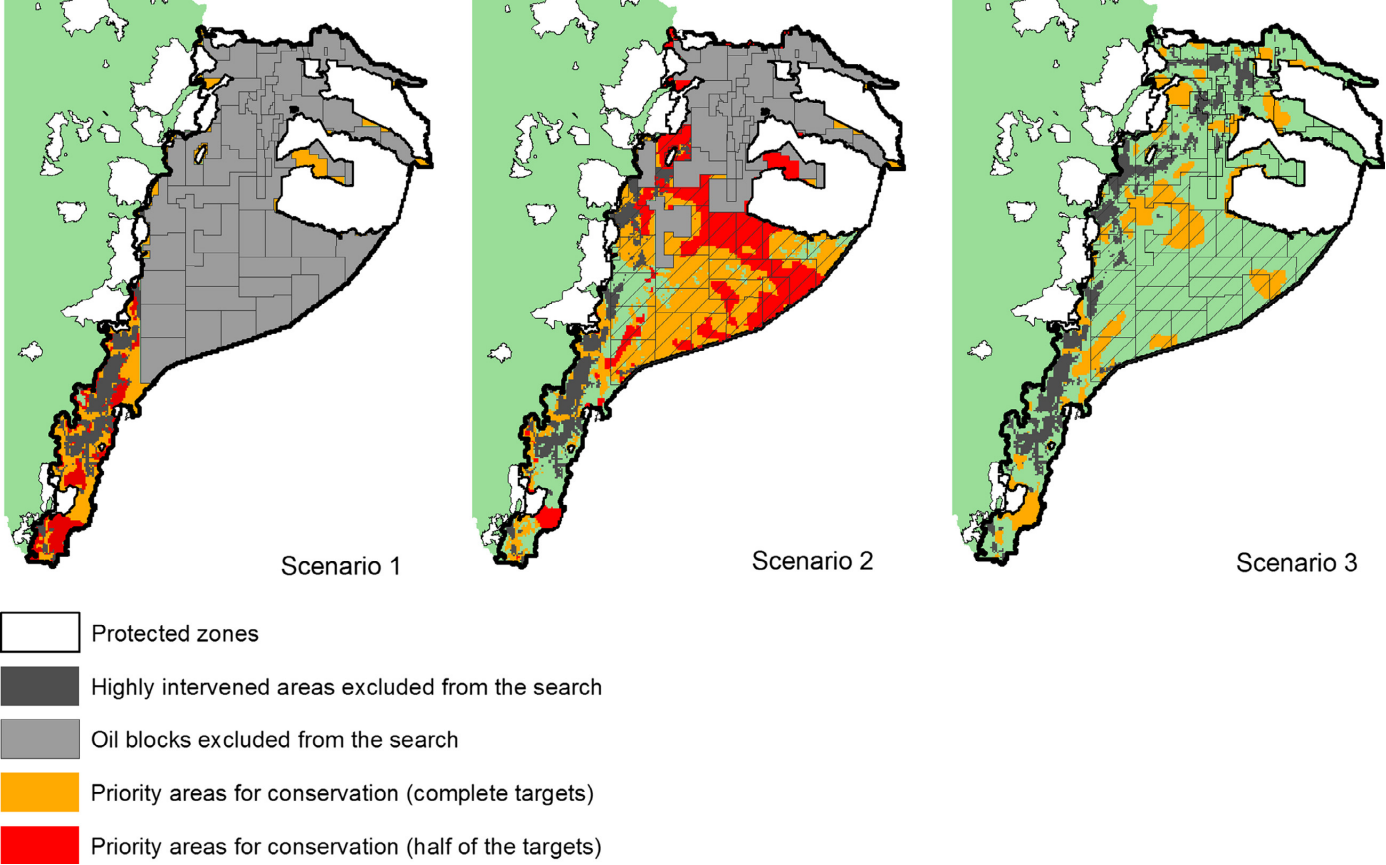
Priority conservation areas in Ecuadorian Amazon selected by Marxan in three different scenarios. Scenario 1: priority areas were searched outside all oil blocks. Scenario 2: priority areas within southern blocks were considered as conservation options. Scenario 3: priority areas were searched across the Ecuadorian Amazon, even inside operative and southern oil blocks. Priority areas identified with half of targets are showed in red, whereas those identified with complete targets are in orange. Notice that priority areas selected with half of targets are contained in the solutions resulting from using complete targets.

In the first scenario, we searched for priority areas in sites that are not affected by present or future oil extraction and show low environmental impact (Fig. 3, Scenario 1). However, these conditions left only a small proportion of the Ecuadorian Amazon (~16%) eligible, mainly located in the south. As a result, the algorithm failed to achieve the defined conservation targets for 45% of the biodiversity indicators even when selecting the majority of PUs available. Thus, we reran the first scenario reducing conservation targets to half of what was originally planned, which allowed identifying a reasonable amount of priority areas. However, these priority areas were again not representative of the Ecuadorian Amazon biodiversity, as Marxan could not reach conservation targets for 11% of the biodiversity indicators. Thus, conservation targets are impossible to achieve in this first scenario (i.e., if prioritization is limited to areas outside of oil blocks).

In the second scenario, we identified priority areas assuming that it is still possible to reserve important sites for conservation within oil blocks that are for bidding in the southern Amazon (Fig. 3, Scenario 2). As a result, priority areas achieved a better representation of biodiversity (93% of targets accomplished). However, Marxan was forced to select almost all the extent (87%) of the southern oil blocks in order to meet the targets. Applying this scheme would imply preserving most blocks from the *XI Ronda Petrolera*, which seems not feasible based on the economic interests of the national government. Thus, we used half of the original targets to rerun this second scenario to provide a more feasible proposal, although less satisfactory from a conservational point of view. The new result presents priority areas that overlap with 33% of the southern oil blocks, while targets were achieved for almost all biodiversity indicators (100% for ecosystems and 99% for species). Finally, the third scenario presents priority areas that coincide with all blocks, including both the operative and the southern blocks (Fig. 3, Scenario 3). In this scenario, Marxan achieved the complete targets for all species and ecosystems and the priority areas occupied 18% of the oil blocks across the Ecuadorian Amazon.

### How important is each oil block for biodiversity conservation?

**Figure 4:**
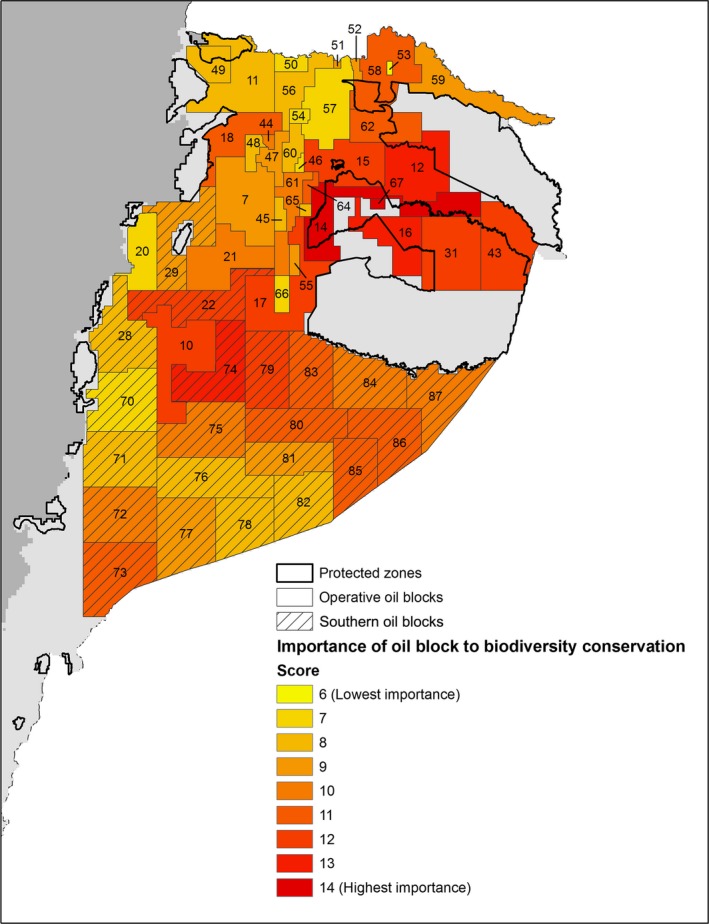
Importance of oil blocks for biodiversity conservation in the Ecuadorian Amazon. The importance index was computed taking into account species (including endemisms and threatened species) and ecosystem diversity, natural vegetation, priority conservation areas for better biodiversity representation, and current protected zones.

From the ranking of importance of oil blocks, four operative blocks (12, 14, 16, and 67) and one southern block (74) resulted with the highest importance for the conservation of biodiversity (Fig. 4, Table S3 in Appendix S3). These blocks are located in the central area of the Ecuadorian Amazon and close to the Yasuní National Park.

## Discussion

Despite its relatively small size (283,560 km^2^), Ecuador is the country with the largest absolute area covered by operative oil blocks (in extraction) in the western Amazon Basin; more than Bolivia, Colombia, Peru, and Venezuela (RAISG, 2012; Finer et al. 2015). Operative oil blocks already occupy a third (32%) of the Ecuadorian Amazon, and the *XI Ronda Petrolera* is bidding an additional 36%, which will duplicate the surface covered by oil concessions in the region. Thus, at the end of this bidding process, Ecuador will have the great majority of its Amazon (~68% [68,200 km^2^]) compromised by oil operations.

Currently, protected and UA cover 22% of the Ecuadorian Amazon, which seems optimal for the conservation of biodiversity. This percentage is particularly impressive, considering that the Convention of Biological Diversity established 17% as the minimum area to be protected within an ecological region (UNEP/CBD, 2011). However, according to our analysis, only 16% of the Ecuadorian Amazon is actually covered by portions of protected and UA free of oil extraction. For example, despite its “strict” protection category (II IUCN; Dudley 2008) and its global priority for conservation (Bass et al. 2010), the Yasuní National Park has 45% of its surface covered by oil blocks. Moreover, 4% of UA overlap with oil blocks. This is a small percentage, but represents an inconsistence in environmental policy, as UA were created to protect biodiversity and social values from any extractive activities (Melo et al. 2009). Then, how can oil mining be emplaced in such sensitive areas? Ecuador's Constitution (Constitución del Ecuador, 2008) allows lifting the prohibition to oil exploitation after a declaration of national interest by Presidential Decree. As result of this policy, almost one‐third of the protected zones in the Ecuadorian Amazon have operative oil blocks within their limits, which surely is affecting the effectiveness of these reserves in protecting biodiversity (Canady and Rivadeneyra 2001; Fiori and Zalba 2003; Suárez et al. 2009; McCracken and Forstner 2014).

In contrast to what happens with operative oil blocks, oil blocks from *XI Ronda Petrolera* show little overlap with protected zones, mostly because there are only three very small PA in the southern Ecuadorian Amazon (El Quimi Biological Reserve, El Cóndor Biological Reserve, and El Zarza Wildlife Reserve). This lack of protection in the southern Amazon makes this region even more vulnerable to the expansion of the oil industry and other extractive activities. In fact, it has been shown that the biodiversity of this region does not have an efficient protected area network to guarantee its long‐term persistence (Cuesta‐Camacho et al. 2006; Lessmann et al. 2014). It should also be noted that an important amount of these southern oil blocks coincide with titled lands of indigenous peoples (Melo et al. 2007). In response, indigenous groups have confronted Government plans, arguing that the new policy will sacrifice ancestral and pristine territories (CONFENIAE and CONAIE 2012; El Comercio, 2012).

In addition to their overlap with protected and UA, operative oil blocks coincide with the potentially most diverse areas for all taxonomic groups, located in the northwest Ecuadorian Amazon, in the transition of Andean‐Amazon ecosystems. Specifically, 99.8% of the species richness center (all five groups) overlaps with oil blocks and deforested areas. As consequence, several species may have become vulnerable and locally extinct in these areas, which are predicted as climatically suitable, but that currently present high habitat loss and strong environmental impacts related to activities of the oil industry. Potential species richness is particularly high in the northwest of the Ecuadorian Amazon because, according to our predictor variables, this region presents high levels of precipitation, which is positively correlated with the richness of birds, amphibians, and plants in the tropics (Herzog et al. 2012). Thus, this pattern is similar to those found by other studies for birds (Ridgely and Greenfield 2007; Bass et al. 2010), amphibian (Bass et al. 2010), and heliconiine butterflies (Rosser et al. 2012). Our richness center, however, seems more restricted than reported before, probably because we used SDMs instead the broader geographic ranges. The question that stands is, as the identification of these species richness centers is based on SDMs and species ranges developed using historical data (mostly museum specimen records), what is the real situation of species in these areas? Are they still striving to survive in the periphery of the most environmentally affected sites? This is a question that can only be answered gathering contemporary field data on species distributions and abundances.

It is also important to mention that as the Amazon has many areas that remain remote or difficult to access, the species occurrence data used had a strong sampling bias. In some cases, this bias may result in too restricted SDMs and false species absences that could affect the accuracy of species richness maps and the design of priority areas for conservation. Still, other types of data available for highly diverse areas, such as observed points or geographic ranges, usually produce greater errors in the species distribution mapping than SDMs (Rondinini et al. 2006; Carvalho et al. 2010; Bombi et al. 2011).

Inclusion of ecosystem data as biodiversity indicator intended to provide a broader picture of the potential vulnerability of Amazon biodiversity, providing robustness to the analysis against sampling bias, regarding both species and geographic coverage. In total, 12 of the 25 Amazon ecosystems have their ranges on highly impacted lands or oil blocks, and six are not covered by any protected area. Combined, the results from the analysis of species richness centers and ecosystems suggest that the Ecuadorian Amazon is particularly vulnerable to losses in biodiversity, because peaks of species diversity and a high proportion of ecosystems coincide spatially with oil blocks.

In view of the vulnerability of biodiversity in the Ecuadorian Amazon, we searched for priority areas that, given their high biodiversity representation, should be protected from oil exploitation. The priority areas found in the first scenario (outside all oil blocks), together with the current protected zones, showed a very low representation of biodiversity. This failed exercise revealed that it is not possible to have an adequate representation of species and ecosystems in well preserved areas outside oil blocks. Additionally, protection of these priority areas would be dubious, at best, given that most of the integrity of southern Amazon not covered by oil blocks is already compromised by 166 mining concessions, mostly for copper and gold (Unidad de Gestión Territorial, 2011).

In the second scenario, we allowed defining priority areas coinciding with southern oil blocks and considering the half of original targets in order to present a more feasible result. Although these priority areas in the southern Amazon are not located in the Amazon's most diverse center, they have a high conservation value because they complement the biodiversity protected by current protected zones. Implementation of these priority areas would provide protection to the high diversity of Amazonian species and ecosystems in areas of low environmental impact. Avoiding oil exploitation in these areas should be considered an alternative for future conservation of Amazonian biodiversity in Ecuador.

Discarding the exploitation of southern blocks implies losses in revenues. However, this proposal might be both reasonable and feasible because the reduction of area compromised by oil blocks would not be dramatic (67% of southern block area will remain). Furthermore, oil reserves in these blocks are limited, and have been estimated to last for only 20 years of extraction (Secretaría de Hidrocarburos del Ecuador, 2013); this situation coupled with the sustained reduction on oil barrel prices during 2015 and 2016 (Barchart Market Data, 2016), lowers the value of such extraction even more. In turn, sustainable ecosystem services may contribute to reach similar or even greater economic profits without threatening biodiversity (e.g., ecotourism, biocommerce, discovery of pharmaceuticals, among others). Enhancing the importance of these sustainable economic activities is especially appropriate in line with the “Good Living” (“Buen Vivir”), an innovative concept introduced in the Constitution of Ecuador to promote social and economic development under the scope of a “harmonious coexistence with nature” (SENPLADES, 2009). Finally, although in this second scenario the targets were reduced to half the original targets, which means a smaller contribution to the conservation of the species and ecosystems, we consider that this proposal is a conciliatory option between Ecuadorian's Amazon biodiversity conservation and the economic needs of the country.

Despite the conciliatory option presented in the second scenario, it is likely that the southern oil blocks will be adjudicated in the future if no actions are taken urgently. In this context, our third scenario aimed to highlight priority areas across the entire Ecuadorian Amazon, including all kinds of blocks. Although these priority areas potentially harbor a higher representation of biodiversity than the previous scenarios, the conservation of species and ecosystems would be less efficient, because most priority areas will have to coexist with extractive activities and their negative impacts.

In the context of the third scenario, we analyzed together the information about species and ecosystem richness, and natural vegetation to identify oil blocks of special importance for biodiversity conservation in the Ecuadorian Amazon. Southern oil blocks of high importance seem good candidates for applying strategies of conservation and no exploitation, although the failure of the Yasuní‐ITT Initiative is not a good precedent. Still, oil reserves in the southern blocks are less important than those in the ITT, and therefore the lost oil revenues would be smaller if the Government decides not to exploit them.

In the case of the most important blocks for conservation that are currently operative, to stop or to limit oil extraction is probably virtually impossible to implement. Nonetheless, we stress the importance of ensuring careful operations in these blocks, especially considering that they are located within PA (i.e., the Yasuní National Park). Finer et al. (2013) published a compendium of best practices for oil extraction that, if incorporated by oil companies into their exploration and exploitation operations, could significantly decrease the negative effects on the environment. Proposed practices include, among others, the substitution of new‐road construction by the use of fluvial and aerial transport, and the reduction of the right‐of‐way in wells and pipelines to decrease deforestation. In addition, we consider that the following practices are important in the Ecuadorian context:

- To establish more control points in existing access roads to prevent the entry of unauthorized personnel; this measure will reduce colonization, deforestation, traffic of wildlife, and timber extraction (Rosenfeld et al. 1997).
- To transfer management of control points from oil companies to governmental or independent control agencies. This measure will reduce the chance that access to areas suffering oil spills is restricted by companies avoiding liability.
- To implement concepts such as “Net Positive Impact” (Olsen et al. 2011), in which negative impacts are compensated through conservation activities that are at least equal in value to the impacts that cannot be avoided.

In the Ecuadorian Amazon there are good examples of the implementation of careful operations, such as the exploitation of Block 10, known as Villano Block, in Pastaza province, which we identified among those blocks of high importance for conservation in the Ecuadorian Amazon. In this block, the managing company flies helicopters to the drilling platform instead of building roads, cleared a narrow path for the pipeline, and re‐injects the production water back into the ground (Williams 1999). However, other experiences have been less positive and generate concern about the future of the biodiversity in the Ecuadorian Amazon. Recently, a wide road in Block 31 (located 80% within the Yasuní National Park) was finished by Petroamazonas, the State company, transgressing the environmental impact study and the approved environmental license (Finer et al. 2014).

Finally, in order to offer the proper guidance and recommendations to planners and decision‐makers, it is important to highlight some methodological limitations of our analyses. As mentioned before, SDMs could generate false species absences due to the bias sampling, missing the opportunity to protect other valuable areas for the biodiversity. On the other hand, false species presences generated in areas climatically suitable but with high habitat loss and degradation could lead to the identification of nonrepresentative priority areas. Although we addressed some of these limitations (e.g., excluding the highly intervened areas from the prioritization analysis), we recommend that any initiative for protecting the identified priority areas should first conduct proper field validations and rapid biological inventories. It is also important to reduce the bias in the current species databases, increasing the efforts in building up natural history collections and making them accessible (Fajardo et al. 2014), especially for the most remote areas of the Ecuadorian Amazon.

## Conclusions and Final Considerations

This study presents an updated picture of current and future oil exploitation in the Ecuadorian Amazon, as well as a set of conservation proposals useful for the development of environmental policies in this biodiverse region. According to our results, the current network of PA in the Amazon cannot adequately protect the diversity of species and ecosystems against the development of the oil industry and other human threats. Given the limited conservation options in the northern Amazon because of the long‐established oil exploitation, preserving the southern Amazon becomes essential to improve the protection of Amazonian species and ecosystems. Despite their overall lower richness, priority areas identified in the southern Amazon complement current PA, allowing the representation of almost all biodiversity indicators in the region. In addition, some of these biodiversity indicators include a number of species and ecosystems distributed only in the southern Amazon, making these areas irreplaceable. Finally, if we also consider that the southern Amazon holds large extensions of pristine forest and precious cultural diversity, its conservation importance becomes incalculable. Unfortunately, despite this importance, the expansion of the oil industry in the southern Amazon has not received as much attention as the exploitation of Yasuní‐ITT block.

It has been established that the Ecuadorian government will make an important expansion of the oil industry across the Amazon. Whether this expansion is compatible with the conservation of biodiversity depends on the implementation of immediate conservation measures, based on a reassessment of conservation priorities and conservation actions on the ground. Given the current plans of the government to exploit the Yasuní‐ITT block and to expand the oil map to the south of the Ecuadorian Amazon, it is challenging to propose conservation alternatives for the region. However, the Ecuadorian Government has the obligation and the means to find concrete solutions to help the country meet its economic needs without allowing uncontrolled extraction of nonrenewable natural resources and the affectation of indigenous peoples (De la Bastida 2009). The revenues that will be perceived by expanding the oil industry in southern Ecuador are planned to be used for further investments toward economic diversification. However, in the last four decades of oil extraction economic diversification has not taken place (Larrea et al. 2010). In addition, future oil exports in Ecuador are constrained by limited reserves and, currently, by a steep decrease in oil prices, while the environmental impact of this activity in highly sensitive areas remains critical (Larrea 2013).

Finally, conservation alternatives presented in our study are also relevant for the preservation of the western Amazon. In 2008, Finer et al. reported that ~688,000 km^2^ of oil blocks in the western Amazon are causing a large impact to the biodiversity and the indigenous peoples across the region. Now, given the current expansion of the oil industry in the Ecuadorian Amazon as well as in the Colombian, Bolivian, and Peruvian Amazon (Finer and Orta‐Martínez 2010; Agencia Nacional de Hidrocarburos, 2014; Finer et al. 2015), the environmental and social impacts of the oil industry in the western Amazon are likely to worsen. In this context, studies such as ours should be replicated across the western Amazon, with the aim of updating information about the conflicts between oil extraction and biodiversity conservation, as well as coordinating efforts for the preservation of this important region.

## Conflict of Interest

None declared.

## Supporting information


**Appendix S1.** Extended methods.

**Appendix S2.** List of biodiversity indicators.

**Appendix S3.** Oil block importance index.
