# Supplementary material for: Large expansion of oil industry in the Ecuadorian Amazon: biodiversity vulnerability and conservation alternatives
Source: Ecol Evol. 2016 Jun 24;6(14):4997–5012. doi: 10.1002/ece3.2099 (PMC4979723; doi:10.1002/ece3.2099)
Supplement: Supplementary file 1 — Appendix S1. Extended methods. [file ECE3-6-4997-s001.docx]

**Supporting Information**

Appendix S1 Extended Methods

1. **Species data**

Species occurrences were obtained from specimen databases of the following museums: Missouri Botanical Garden Tropicos Database, American Museum of Natural History; Academy of Natural Sciences of Drexel University; The University of Colorado Museum of Natural History; Delaware Museum of Natural History; Denver Museum of Nature & Science; Museum of Natural History University of Kansas; Natural History Museum of Los Angeles County; Louisiana State University Museum of Natural Science; Museum of Comparative Zoology-Harvard University; Michigan State University Museum; The Museum of Vertebrate Zoology at Berkeley; Oklahoma Museum of Natural History; Royal Ontario Museum; Santa Barbara Museum of Natural History; San Diego Natural History Museum, Alabama Museum of Natural History; The University of Arizona Museum of Natural History; Museum of Zoology, University of Michigan; Museum of the University of Nebraska State Museum; Smithsonian Institution National Museum of Natural History; University of Washington Burke Museum of Natural History and Culture; Yale Peabody Museum of Natural History; and Museo de Zoología de la Pontificia Universidad Católica del Ecuador. Records from [Rosser et al. (2012](#_ENREF_9)) were also used.

The species included represent 51.2% of amphibians, 53.4% of birds, 47.8% of medium and large terrestrial mammals, 6.4% of vascular plants, and a small representation of butterflies that are reported for the Ecuadorian Amazon.

We checked the locality points and we corrected errors in the georreferences using specialized literature [8-11], experts’ knowledge in each taxonomic group, and detailed geographic information for Ecuador (such as the topographic maps for IGM; etc.) [12]. This high quality standard in occurrence data forced us to model only a partial representation of the species diversity in the Ecuadorian Amazon.

1. **Species distribution models**

Given the high number of species in the study, and the possibility that each one would be optimally modeled with a different set of variables, we did not remove any of the 19 variables. The number of background points was set to 10,000 and drawn randomly across the study area. While using the same background for species from different geographic origins may artificially increase AUC (Area Under de Curve) values ([Royle et al., 2012](#_ENREF_10)), we were not interested in the fine details of the individual models’ accuracy, but rather in creating the best possible models to be used by the site-selection algorithm. Accordingly, we discarded species’ SDMs that had AUC values below 0.7 ([Elith and Leathwick, 2007](#_ENREF_4)). We reclassified the 0–1 suitability map into binary (presence/absence) maps using the Maximum Training Sensitivity Plus Specificity threshold ([Liu et al., 2013](#_ENREF_6)). Finally, to obtain more accurate approximations of the species distributions, we used information found in the literature ([Ridgely and Greenfield, 2007](#_ENREF_8), [Tirira, 2007](#_ENREF_12), [AmphibiaWeb, 2012](#_ENREF_1)) to remove areas of over-prediction and areas beyond geographic barriers that likely preclude species dispersal.

**3. Marxan settings**

We used Marxan ([Ball et al., 2009](#_ENREF_3)) to identify priority conservation areas. Marxan’s simulated annealing algorithm selects a set of planning units (PU) that meets the predefined conservation targets while minimizing the total cost of all PUs included in the reserve system ([Ardron et al., 2008](#_ENREF_2)). ‘Total cost’ is defined as:

∑ PUs Cost + BLM ∑ PUs Boundary +∑ Targets Penalty

where summation of PUs cost represents some measure of the cost of including a PU (e.g., purchase value), PUs boundary summation is a cost related to the length of the solution perimeter (agglomerate vs. disperse solution, tuned with the Boundary Length Modifier, BLM), and the targets penalty is the costs imposed for failing to meet the targets.

We used a BLM value of 0.01 that allows generating a reserve network with low fragmentation ([Stewart and Possingham, 2005](#_ENREF_11)). The cost of each PU was equated to its environmental impact, as defined by [McPherson et al. (2008](#_ENREF_7)) and [Lessmann et al. (2014](#_ENREF_5)). This method consists of developing an Environmental Risk Surface (ERS) with the *Protected Area Tools* for ArcMap 9.3 (http://gg.usm.edu/pat/), combining information about the intensity and distance of influence of environmental risks. A risk element is defined as any aspect that has a negative influence on a conservation feature. The intensity value summarizes the relative level of threat that the risk element poses to biodiversity on a 0–100 scale, with 100 being the strongest risk. The influence distance represents the maximum distance at which the element has a negative impact on biodiversity. Within this distance we chose a linear decreasing function to simulate the decline of intensity starting at the location of the risk element. Based on the available data, we selected the following risk elements: human population density, agriculture and cattle ranching, mining (other than oil extraction), oil wells, roads, and airports. Each risk element was assigned an intensity value and an influence distance based in the literature and our best judgment (Table 1). This process yielded a raster layer where each pixel had an impact value between 0–100.

All scenarios were analyzed running Marxan with the simulated annealing method, followed by iterative improvement, using 10^8^ iterations and 100 replicates. The priority areas identified in each scenario were evaluated in terms of the achievement of conservation targets, extent, and spatial coincidence with other conflicts, such as mineral mining, which are not the focus on this study but may compromise the feasibility of the priority areas proposals.

**Table S1. Intensity and influence distances assigned to different risk elements used to create the environmental risk surface.**

| **Risk element** | **Class** | **Intensity**  **(0–100)** | **Influence distance** |
| --- | --- | --- | --- |
| Roads | 4-lane highway | 50 | 1,000 m |
|  | 2-lane highway | 25 |  |
|  | Track | 17 |  |
|  | Railroad | 17 |  |
|  | Dirt roads | 10 |  |
| Human population density | – | Normalized between 0 and 100 according to the logistic function | Scaled from 5 km from the lower intensity |
| Airports | – | 70 | 2,000 m |
| Agriculture and cattle raising | Monoculture plantations and cattle raising | 50 | 2,000 m |
|  | Mixed crops | 40 | 1,000 m |
|  | Forestry crops and crops in protected forests | 30 | 1,000 m |
|  | Shrimp farms | 50 | 2,000 m |
| Mining industry | Mining concessions in exploitation | 50 | 10,000 m |

**References**

AMPHIBIAWEB. 2012. *Information on amphibian biology and conservation* [Online]. Berkeley, California. Available: http://amphibiaweb.org/.

ARDRON, J. A., POSSINGHAM, H. P. & KLEIN, C. J. 2008. *Marxan Good Practices Handbook,* Vancouver, Pacific Marine Analysis and Research Association.

BALL, I. R., POSSINGHAM, H. P. & WATTS, M. E. 2009. Marxan and relatives: Software for spatial conservation prioritisation. *In:* MOILANEN, A., WILSON, K. A. & POSSINGHAM, H. P. (eds.) *Spatial conservation prioritisation: Quantitative methods and computational tools.* Oxford, UK: Oxford University Press.

ELITH, J. & LEATHWICK, J. 2007. Predicting species distributions from museum and herbarium records using multiresponse models fitted with multivariate adaptive regression splines. *Diversity and Distributions,* 13**,** 265-275.

LESSMANN, J., MUÑOZ, J. & BONACCORSO, E. 2014. Maximizing species conservation in continental Ecuador: a case of systematic conservation planning for biodiverse regions. *Ecology and Evolution,* 4**,** 2410-2422.

LIU, C., WHITE, M. & NEWELL, G. 2013. Selecting thresholds for the prediction of species occurrence with presence-only data. *Journal of Biogeography,* 40**,** 778-789.

MCPHERSON, M., SCHILL, S., RABER, G., JOHN, K., ZENNY, N., THURLOW, K. & SUTTON, A. H. 2008. GIS-based modeling of Environmental Risk Surfaces (ERS) for conservation planning in Jamaica. *Journal of Conservation Planning,* 4**,** 60-89.

RIDGELY, R. & GREENFIELD, P. J. 2007. *Aves del Ecuador. Volumen II Guía de Campo,* Quito, Fundación Jocotoco.

ROSSER, N., PHILLIMORE, A. B., HUERTAS, B., WILLMOTT, K. R. & MALLET, J. 2012. Testing historical explanations for gradients in species richness in heliconiine butterflies of tropical America. *Biological Journal of the Linnean Society,* 105.

ROYLE, J. A., CHANDLER, R. B., YACKULIC, C. & NICHOLS, J. D. 2012. Likelihood analysis of species occurrence probability from presence-only data for modelling species distributions. *Methods in Ecology and Evolution,* 3**,** 545-554.

STEWART, R. R. & POSSINGHAM, H. P. 2005. Efficiency, costs and trade-offs in marine reserve system design. *Environmental Modeling and Assessment,* 10**,** 203–213.

TIRIRA, D. 2007. *Guía de Campo de los mamíferos del Ecuador,* Quito, Ediciones Murciélago Blanco, Publicación especial sobre los mamíferos del Ecuador 6.
